# Increased CD95^+^ or CD160^+^ double-negative T-cell subsets are associated with incomplete immune reconstitution in ART-treated people living with HIV

**DOI:** 10.3389/fimmu.2026.1806055

**Published:** 2026-04-16

**Authors:** Jingli Peng, Yanyan Li, Jiale Xi, Zihao Xia, Ping Luo, Shuo Yang, Lianfeng Lu, Chen Chen, Rentian Cai, Qin Wang, Hongxia Wei

**Affiliations:** Department of Infectious Diseases, The Second Hospital of Nanjing, Affiliated to Nanjing University of Chinese Medicine, Nanjing, Jiangsu, China

**Keywords:** CD160, CD95 (Fas/APO-1), double-negative T cells, immune activation, incomplete immune reconstitution, people living with HIV

## Abstract

**Objective:**

To investigate the expression patterns of CD95 and CD160 on double-negative T (DN T) cells and their association with incomplete immune reconstitution in people living with HIV.

**Methods:**

In this cross-sectional study, people living with HIV (PLWH) receiving long-term antiretroviral therapy (ART) with sustained viral suppression were classified as immunological non-responders (INR) or immunological responders (IR). Flow cytometry was used to characterize double-negative T (DN T) cells and their CD95^+^ and CD160^+^ subsets. Associations between DN T-cell phenotypes and INR were evaluated using univariable and multivariable logistic regression analyses, with odds ratios (ORs) calculated per 10% increase in cell percentages. Receiver operating characteristic (ROC) curves were used to assess discriminatory ability.

**Results:**

The frequency of DN T cells was significantly higher in the INR group than in the IR group (P = 0.022). The frequencies of CD95^+^ DN T cells and CD160^+^ DN T cells were increased in INR participants (P < 0.001 and P = 0.003, respectively) and were negatively correlated with CD4^+^ T-cell counts (CD95: r = −0.447, P < 0.001; CD160: r = −0.281, P = 0.008). After adjustment for age, ART duration, and baseline CD4^+^ T-cell counts, multivariable analysis showed that CD95^+^ DN T cells and CD160^+^ DN T cells were independently associated with INR. ROC analysis showed moderate discriminatory ability for CD95^+^ DN T cells (AUC = 0.788), whereas CD160^+^ DN T cells demonstrated lower discriminatory performance (AUC = 0.687). Adding DN T-cell phenotypic parameters to a baseline clinical model modestly improved discriminatory performance.

**Conclusion:**

Incomplete immune reconstitution in PLWH is associated with phenotypic alterations of DN T cells characterized by increased CD95 and CD160 expression. These findings provide a phenotypic characterization of DN T-cell alterations associated with immune dysregulation in INR and may offer insights for further mechanistic investigation.

## 1 Introduction

Despite durable virological suppression achieved with combination antiretroviral therapy (ART), a substantial proportion of people living with HIV (PLWH) fail to achieve adequate immune recovery. These individuals are commonly referred to as immunological non-responders (INR), a condition also described as incomplete immune reconstitution. This condition is characterized by persistently low CD4^+^ T-cell counts despite effective viral suppression and is frequently accompanied by sustained immune activation and an increased risk of opportunistic infections and non-AIDS-related complications (1). However, the mechanisms underlying incomplete immune reconstitution remain complex and incompletely understood. Moreover, early identification of individuals at risk remains challenging, and effective intervention strategies are limited, as no specific pharmacological therapies are currently available once INR has developed (2). Therefore, improving the understanding of immunological alterations associated with INR remains an important research priority.

Immune reconstitution involves both the quantitative recovery and functional restoration of multiple T-cell subsets, and the dynamic balance among these populations is essential for maintaining immune homeostasis. Among these subsets, double-negative T (DN T) cells (CD3^+^CD4^-^CD8^-^) account for approximately 1%–5% of peripheral T cells in healthy individuals. DN T cells are heterogeneous in origin and may arise during thymic development or through peripheral differentiation of CD4^+^ or CD8^+^ T cells that lose coreceptor expression under conditions of chronic antigenic stimulation (3). Functionally, DN T cells exhibit considerable plasticity. Previous studies suggest that they can regulate immune responses and limit excessive inflammation (4), while also producing proinflammatory cytokines such as IL-17 and IFN-γ that may contribute to persistent immune activation (5). Accordingly, DN T cells are increasingly recognized as an immunoregulatory T-cell population involved in inflammatory diseases and immune dysregulation.

In recent years, increasing attention has been directed toward the role of DN T cells in HIV infection and incomplete immune reconstitution. During HIV infection, alterations in the frequency and functional characteristics of DN T cells have been associated with disease progression. Acute HIV infection has been reported to influence DN T-cell frequencies, with DN T-cell counts positively correlated with CD4^+^ T-cell counts and negatively correlated with viral load (6). In addition, increased frequencies of activation-associated DN T-cell subsets, including HLA-DR^+^ DN T cells, CD38^+^ DN T cells, and HLA-DR^+^CD38^+^ DN T cells, have been observed during acute infection. Notably, these alterations are not fully normalized even after early initiation of ART, suggesting that HIV-related dysregulation of DN T cells may persist despite effective viral suppression (7). In PLWH with incomplete immune reconstitution, recent studies have reported reduced absolute numbers of circulating DN T cells compared with immunological responders (IR). Moreover, DN T cells from INR individuals show increased expression of activation markers such as CD38 and HLA-DR, together with reduced levels of perforin and granzyme B, and these alterations have been reported to correlate negatively with CD4^+^ T-cell counts (8). These findings suggest that functional alterations of DN T cells may be associated with the immunological abnormalities observed in INR. Furthermore, TCRαβ^+^ DN T cells have been reported to expand in PLWH and to exhibit proinflammatory and cytotoxic characteristics, supporting a potential role of this subset in HIV-associated immune dysregulation (9).

In T-cell-mediated immune regulation, the surface molecules CD160 and CD95 have been implicated in T-cell inhibitory and apoptotic pathways, respectively. Previous studies indicate that during chronic HIV infection, increased CD160 expression on CD8^+^ T cells is associated with features of functional exhaustion and impaired antiviral responses (10, 11). In parallel, activation of the CD95 (Fas) signaling pathway under conditions of persistent immune activation has been linked to increased susceptibility of T cells to apoptosis, potentially contributing to immune homeostasis disruption (12). In addition, CD134 (OX40) has been reported to influence the abundance and functional properties of DN T cells, including by promoting the conversion of CD4^+^ T cells into DN T cells and supporting their proliferation and survival (13, 14).

Notably, although DN T cells have been increasingly implicated in immune dysregulation during HIV infection and incomplete immune reconstitution, the expression patterns of CD160, CD95, and CD134 on DN T cells in PLWH with INR have not been systematically characterized. Therefore, the present study aimed to characterize the expression profiles of CD160, CD95, and CD134 on DN T cells and their subsets in PLWH with INR and to examine their associations with incomplete immune reconstitution. This study provides a more comprehensive phenotypic description of DN T-cell alterations in INR and contributes to a better understanding of the immunological features associated with incomplete immune reconstitution.

## 2 Materials and methods

### 2.1 Study population and materials

#### 2.1.1 Study participants

Participants were adult PLWH who initiated ART from January 2018 onward and were prospectively followed at the outpatient clinic of the Department of Infectious Diseases, The Second Hospital of Nanjing, China. Peripheral blood samples, demographic characteristics, and clinical laboratory data were collected from eligible participants. Baseline measurements were defined as those obtained within 1 month prior to ART initiation to ensure temporal proximity to treatment initiation.

The inclusion criteria were as follows: confirmed diagnosis of HIV/AIDS; age ≥18 years and <80 years. Participants in the INR group (n = 50) were defined by persistently low CD4^+^ T-cell counts <350 cells/μL, whereas those in the IR group (n = 37) were defined by sustained CD4^+^ T-cell counts >500 cells/μL and a CD4^+^/CD8^+^ T-cell ratio >1. Normalization of the CD4^+^/CD8^+^ T-cell ratio is recognized as an indicator of immune restoration, and persistently low ratios are associated with immune activation and increased risk of non-AIDS morbidity and mortality (15, 16). Therefore, both CD4^+^T-cell recovery and CD4^+^/CD8^+^ T-cell ratio normalization were considered in defining immunological responders in this study. All participants in both groups had received ART for more than 4 years and maintained sustained viral suppression, defined as plasma HIV RNA levels <50 copies/mL for more than 3 years.

Exclusion criteria included conditions that could independently cause prolonged CD4^+^ T-cell depletion, such as known primary or secondary immunodeficiency, chronic viral infections, hematological malignancies, or long-term use of immunosuppressive medications (17).

This study was approved by the Medical Ethics Committee of The Second Hospital of Nanjing and was conducted in accordance with ethical standards (approval number: 2025-LS-ky-025).

#### 2.1.2 Sample collection and processing

Peripheral blood mononuclear cells (PBMCs) were isolated using standard Ficoll-Paque density gradient centrifugation as previously described (18). PBMCs samples used for flow cytometric analyses were collected at the time when participants had achieved sustained virological suppression and were classified as INR or IR. The isolated PBMCs were cryopreserved in freezing medium containing 90% fetal bovine serum (FBS) and 10% dimethyl sulfoxide (DMSO) and stored in liquid nitrogen until analysis, and all procedures were completed within 24 hours of blood collection.

#### 2.1.3 Flow cytometry antibodies

The following fluorochrome-conjugated monoclonal antibodies were used: anti-human CD45 PerCP-Cy5.5 (clone HI30), CD3 APC-Cy7 (SK7), CD4 PE-Cy7 (SK3), CD8 BV605 (RPA-T8), TCRαβ BV510 (IP26), CD160 Alexa Fluor 488 (BY55), CD95 BV421 (DX2), CD134 PE (ACT35), CD38 APC (HB7), and HLA-DR BV711 (L203) (all from BD Pharmingen, USA), along with the corresponding isotype controls.

#### 2.1.4 Other materials

Major reagents used in this study included Ficoll-Paque™ PLUS (Cytiva, USA), RPMI 1640 medium (Gibco, USA), fetal bovine serum (Absin, China), and phosphate-buffered saline (PBS; Gibco, USA). Details of instruments and assay-specific reagents are described in the corresponding methodological sections below.

### 2.2 Methods

#### 2.2.1 Absolute T-lymphocyte counts

EDTA-anticoagulated peripheral blood samples were used for the determination of absolute T-lymphocyte counts. Staining was performed using TruCount absolute counting tubes (BD Biosciences) in combination with multicolor fluorochrome-conjugated antibodies against CD3, CD4, CD8, and CD45. The percentages and absolute counts of CD3^+^, CD4^+^, and CD8^+^ T cells were measured by flow cytometry.

#### 2.2.2 Viral load measurement

Plasma HIV-1 RNA levels were quantified using the Abbott RealTime HIV-1 assay (Abbott Laboratories), with a lower limit of detection of 20 copies/mL.

#### 2.2.3 Flow cytometry

Cryopreserved PBMCs were rapidly thawed in a 37 °C water bath and washed with phosphate-buffered saline (PBS). Cell viability was assessed using trypan blue exclusion, and samples with viability greater than 85-90% were used for subsequent staining and analysis. All samples were processed using the same thawing and staining procedures to minimize potential technical variability. After centrifugation, cells were resuspended in PBS and stained with pre-mixed fluorochrome-conjugated monoclonal antibodies. Samples were incubated for 30 min at room temperature in the dark, washed with PBS, and finally resuspended in PBS for acquisition. Data acquisition was performed using a BD FACSAria™ III flow cytometer (BD Biosciences, USA). Fluorescence compensation was established using single-stained controls, and appropriate isotype controls were included when necessary. To ensure consistency across samples, the same gating strategy was applied throughout the analysis. Data were analyzed using FlowJo software (Tree Star Inc., Ashland, OR, USA).

**Figure 1:**
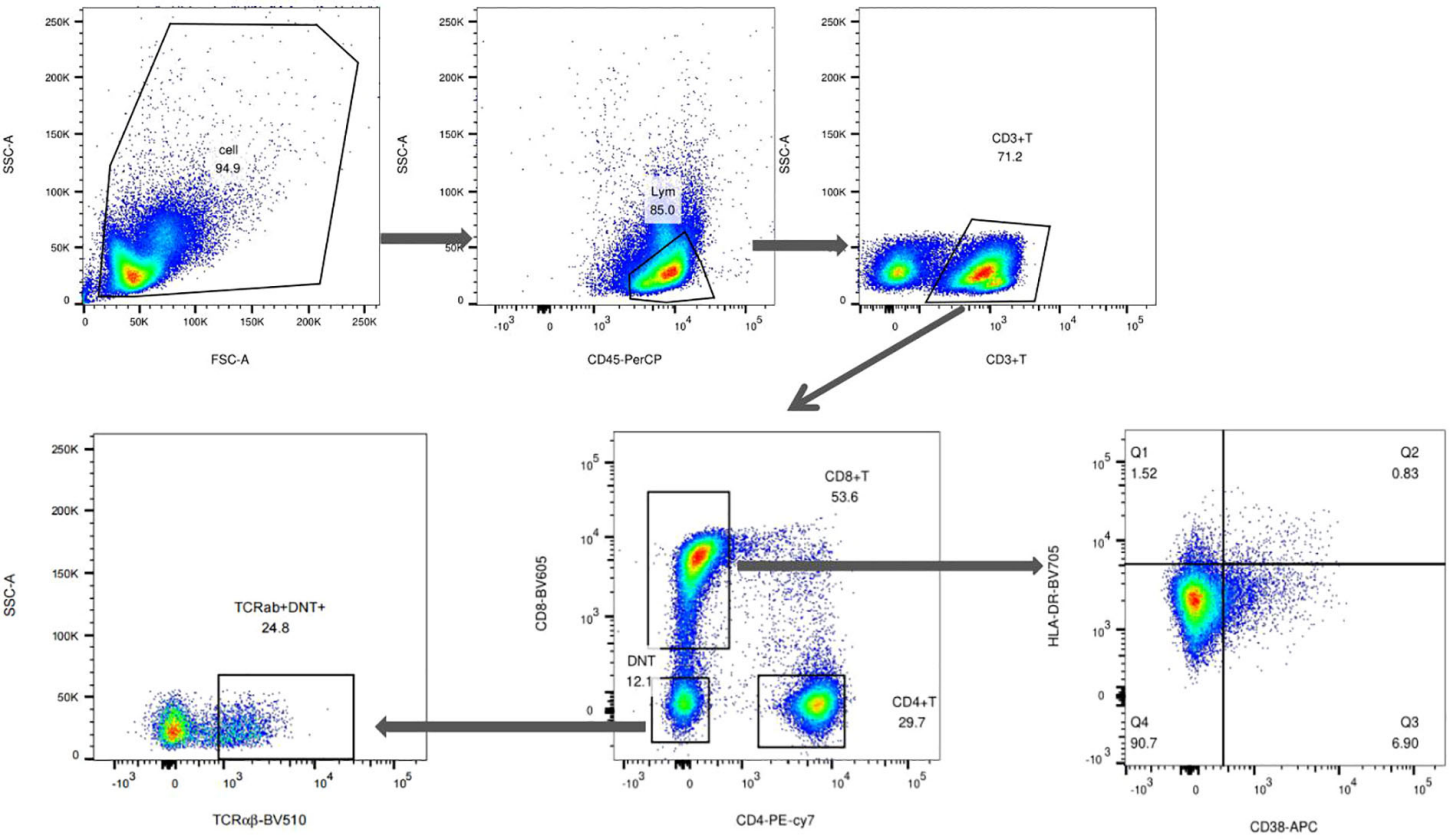
Flow cytometry gating strategy for identification of DN T cells. Peripheral blood mononuclear cells (PBMCs) were first gated on lymphocytes based on forward scatter (FSC-A) and side scatter (SSC-A) characteristics, followed by selection of CD45^+^ cells. CD3^+^ T cells were then identified, and DN T cells were defined as CD3^+^CD4^-^CD8^-^ cells. The TCRαβ^+^ DN T-cell subset was subsequently gated for downstream phenotypic analyses. Systemic immune activation was assessed by the frequency of CD38^+^HLA-DR^+^CD8^+^ T cells. For clarity, representative gating for the major populations is shown, whereas the analysis of additional phenotypic markers was performed within the defined DN T-cell or TCRαβ^+^ DN T-cell gates as described in the Methods section.

For flow cytometric analysis, lymphocytes were first identified based on forward scatter (FSC-A) and side scatter (SSC-A) characteristics and further confirmed by CD45 expression. CD3^+^ T cells were subsequently gated, and DN T cells were defined as CD3^+^CD4^-^CD8^-^ cells. Within this population, TCRαβ expression was used to identify the TCRαβ^+^ DN T-cell subset. Systemic immune activation was evaluated by the frequency of CD38^+^HLA-DR^+^ CD8^+^ T cells. The expression of CD95, CD160, CD134, and activation-associated markers (CD38 and HLA-DR) was analyzed within total DN T cells and the TCRαβ^+^ DN T-cell subset. The detailed gating strategy is shown in **Figure 1**.

### 2.3 Statistical analysis

Data management was performed using Microsoft Excel. Statistical analyses were conducted using SPSS version 22.0 (IBM Corp.), and Figures were generated with GraphPad Prism version 9.5 (GraphPad Software). Continuous variables were assessed for normality. Normally distributed variables were expressed as mean ± standard deviation (SD) and compared using the independent-samples t test, whereas non-normally distributed variables were presented as median (interquartile range, IQR) and compared using the Mann-Whitney U test. Categorical variables were expressed as counts (percentages) and compared using the chi-square test, as appropriate. Spearman’s rank correlation was used for correlation analyses. Logistic regression analysis was performed with immune reconstitution status as the dependent variable (INR = 1, IR = 0). Univariable logistic regression was first applied to evaluate associations between individual variables and INR. In multivariate models, percentages of DN T cells and their subsets were multiplied by 10, and odds ratios (ORs) were expressed per 10% increase for ease of interpretation. To account for potential confounding by clinical variables, multivariable logistic regression analyses were performed including age, ART duration, and baseline CD4^+^ T-cell count as covariates. A stepwise modeling approach was used, with each model including one DN T-cell phenotypic variable and the predefined covariates, to assess independent associations with INR. Receiver operating characteristic (ROC) curve analysis was performed to evaluate the discriminatory ability of individual variables and models for INR using the same transformed variables as in the regression analyses. The area under the curve (AUC) and its 95% confidence interval (95% CI) were calculated. Optimal cutoff values were determined using the Youden index, with corresponding sensitivity and specificity reported. All tests were two-sided, and a P value < 0.05 was considered statistically significant. Numerical results were reported to a maximum of three decimal places and rounded according to standard rounding rules, with P values < 0.001 reported as P < 0.001.

## 3 Results

### 3.1 Baseline and demographic characteristics of INR and IR groups

A total of 87 PLWH were included in this study, comprising 50 individuals classified as INR and 37 as IR. Baseline demographic and clinical characteristics at baseline (within 1 month prior to ART initiation) are summarized in **Table 1**. Participants in the INR group were significantly older than those in the IR group (median age: 57 vs. 43 years, P = 0.029) and had markedly lower baseline CD4^+^ T-cell counts (median: 58 vs. 367 cells/μL, P < 0.001). In addition, the duration of ART was significantly shorter in the INR group than in the IR group (median: 1972 vs. 2619 days, P < 0.001). No significant differences were observed between the two groups in sex, marital status, route of HIV acquisition, baseline viral load, or the presence of coinfections.

**Table 1:** Baseline demographic and clinical characteristics of INR and IR participants.

Variable	INR (n = 50)	IR (n = 37)	P value
Sex, n (%)			0.215
Male	48 (96.0)	33 (89.2)	
Female	2 (4.0)	4 (10.8)	
Age, years, median (IQR)	57 (41-61)	43 (35-58)	0.029
Marital status, n (%)			0.388
Married	20 (40.0)	16 (43.2)	
Unmarried	26 (52.0)	15 (40.5)	
Divorced/Widowed	4 (8.0)	6 (16.2)	
Route of HIV acquisition, n (%)			0.828
Homosexual	32 (64.0)	26 (70.3)	
Heterosexual	13 (26.0)	8 (21.6)	
Other	5 (10.0)	3 (8.1)	
Baseline HIV viral load (copies/mL), median (IQR)	72300 (23600-167500)	35000 (7770-93700)	0.074
< 10^5^ copies/mL, n (%)	27 (54.0)	25 (67.6)	0.343
≥ 10^5^ copies/mL, n (%)	18 (36.0)	8 (21.6)	
Missing baseline VL, n (%)	5 (10.0)	4 (10.8)	
Baseline CD4^+^ T-cell count (cells/µL), median (IQR)	58 (18-125)	367 (288-517)	< 0.001
< 200 cells/µL, n (%)	46 (92.0)	2 (5.4)	< 0.001
≥ 200 cells/µL, n (%)	4 (8.0)	35 (94.6)	
Coinfections, n (%)			
Hepatitis B virus	13 (26.0)	5 (13.5)	0.155
Hepatitis C virus	1 (2.0)	2 (5.4)	0.389
Syphilis	3 (6.0)	6 (16.2)	0.122
ART duration (days), median (IQR)	1972 (1761-2178)	2629 (2137-3403)	< 0.001

### 3.2 Increased DN T cells frequency is associated with immune activation in PLWH with INR

**Figure 2:**
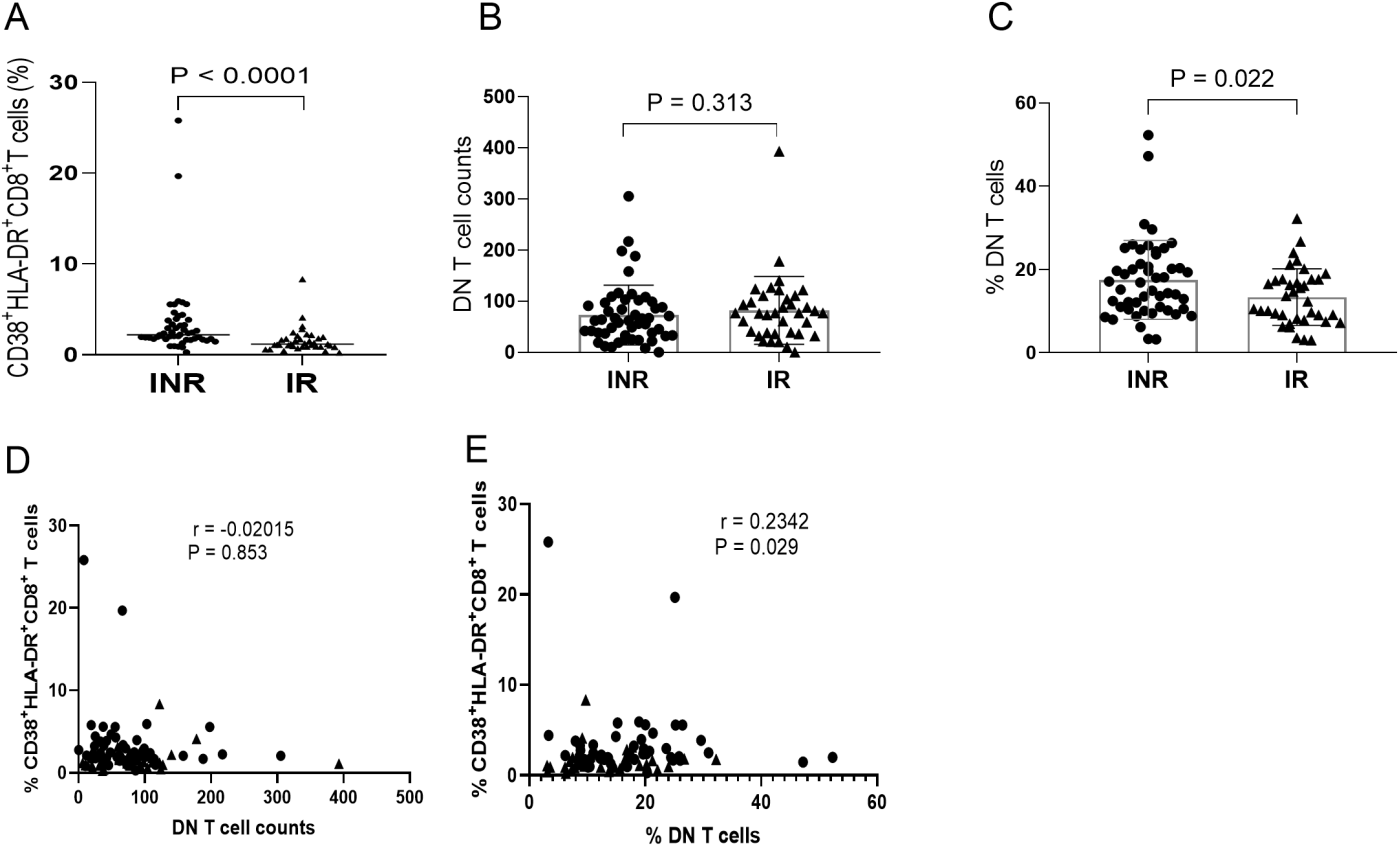
Systemic immune activation and DN T cells in INR and IR individuals. **(A)** Frequency of CD38^+^HLA-DR^+^CD8^+^ T cells in INR and IR groups. **(B)** Absolute counts of DN T cells. **(C)** Frequency of DN T cells. **(D)** Correlation between CD38^+^HLA-DR^+^CD8^+^ T cells and DN T-cell counts. **(E)** Correlation between CD38^+^HLA-DR^+^CD8^+^CD8^+^ T cells and DN T-cell frequency. Each dot represents an individual participant; horizontal bars indicate median with interquartile range (IQR). • INR; ▲ IR. INR, immunological non-responders; IR, immunological responders; DN T cells, double-negative T cells.

Systemic immune activation and the overall characteristics of DN T cells were first evaluated in the INR and IR groups. As shown in **Figure 2A**, the frequency of CD38^+^HLA-DR^+^CD8^+^ T cells was significantly higher in the INR group than in the IR group (P < 0.001). The absolute counts of DN T cells in the INR group showed a decreasing trend compared with the IR group; however, the difference was not statistically significant (P = 0.313; **Figure 2B**). In contrast, the frequency of DN T cells in peripheral blood was significantly higher in PLWH with INR than in those with IR (P = 0.022; **Figure 2C**). To further examine the relationship between systemic immune activation and DN T cells, correlation analyses were performed between CD38^+^HLA-DR^+^CD8^+^ T cells and DN T-cell parameters. The frequency of CD38^+^HLA-DR^+^CD8^+^ T cells showed a negative trend with the absolute counts of DN T cells, although this association did not reach statistical significance (r = −0.207, P = 0.054; **Figure 2D**). In contrast, CD38^+^HLA-DR^+^CD8^+^ T-cell frequency was positively correlated with DN T-cell frequency (r = 0.236, P = 0.028; **Figure 2E**).

### 3.3 Altered phenotypic profiles of DN T cells in PLWH with INR

**Figure 3:**
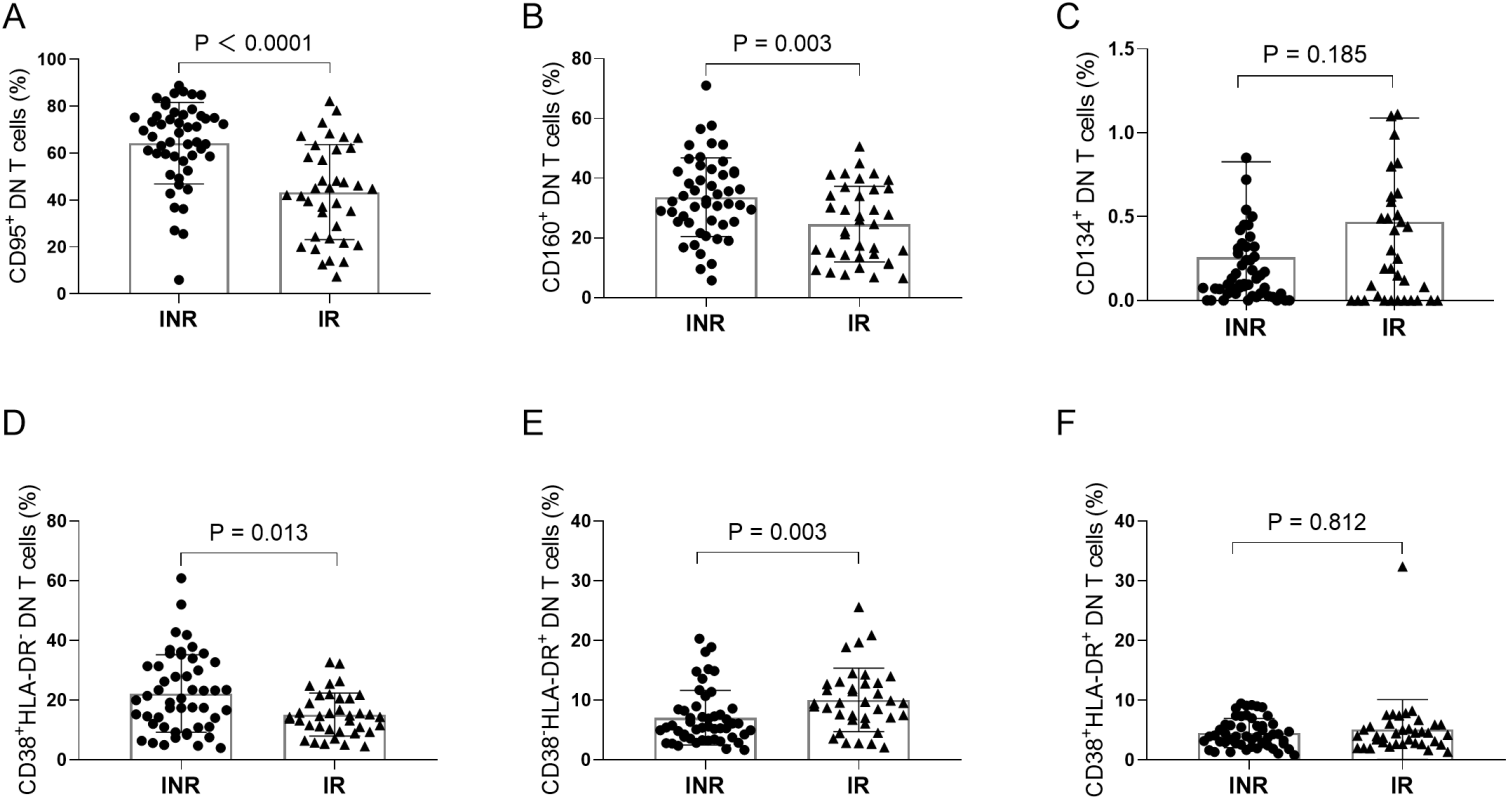
Phenotypic and activation characteristics of DN T cells in INR and IR individuals. **(A–C)** Frequencies of CD95^+^, CD160^+^, and CD134^+^ DN T cells. **(D)** Frequency of CD38^+^HLA-DR^-^ DN T cells. **(E)** Frequency of CD38^-^HLA-DR^+^ DN T cells. **(F)** Frequency of CD38^+^HLA-DR^+^ DN T cells. Each dot represents an individual participant; horizontal bars indicate the median with interquartile range (IQR). • INR; ▲ IR. INR, immunological non-responders; IR, immunological responders; DN T cells, double-negative T cells.

We next evaluated the phenotypic characteristics of DN T cells by analyzing the expression of several surface markers. Compared with the IR group, the frequencies of CD95^+^ DN T cells (P < 0.001) and CD160^+^ DN T cells (P = 0.003) were significantly increased in the INR group (**Figures 3A, B**). In contrast, no significant difference was observed in the frequency of CD134^+^ DN T cells between the two groups (**Figure 3C**). In addition, the frequency of CD38^+^HLA-DR^-^ DN T cells was significantly higher in the INR group than in the IR group (P = 0.013) (**Figure 3D**). Conversely, the frequency of CD38^-^HLA-DR^+^ DN T cells was significantly lower in the INR group (P = 0.003) (**Figure 3E**). No significant difference was detected in the frequency of CD38^+^HLA-DR^+^ DN T cells between the two groups (**Figure 3F**).

### 3.4 Elevated CD95 and CD160 expression on DN T cells is associated with INR

**Figure 4:**
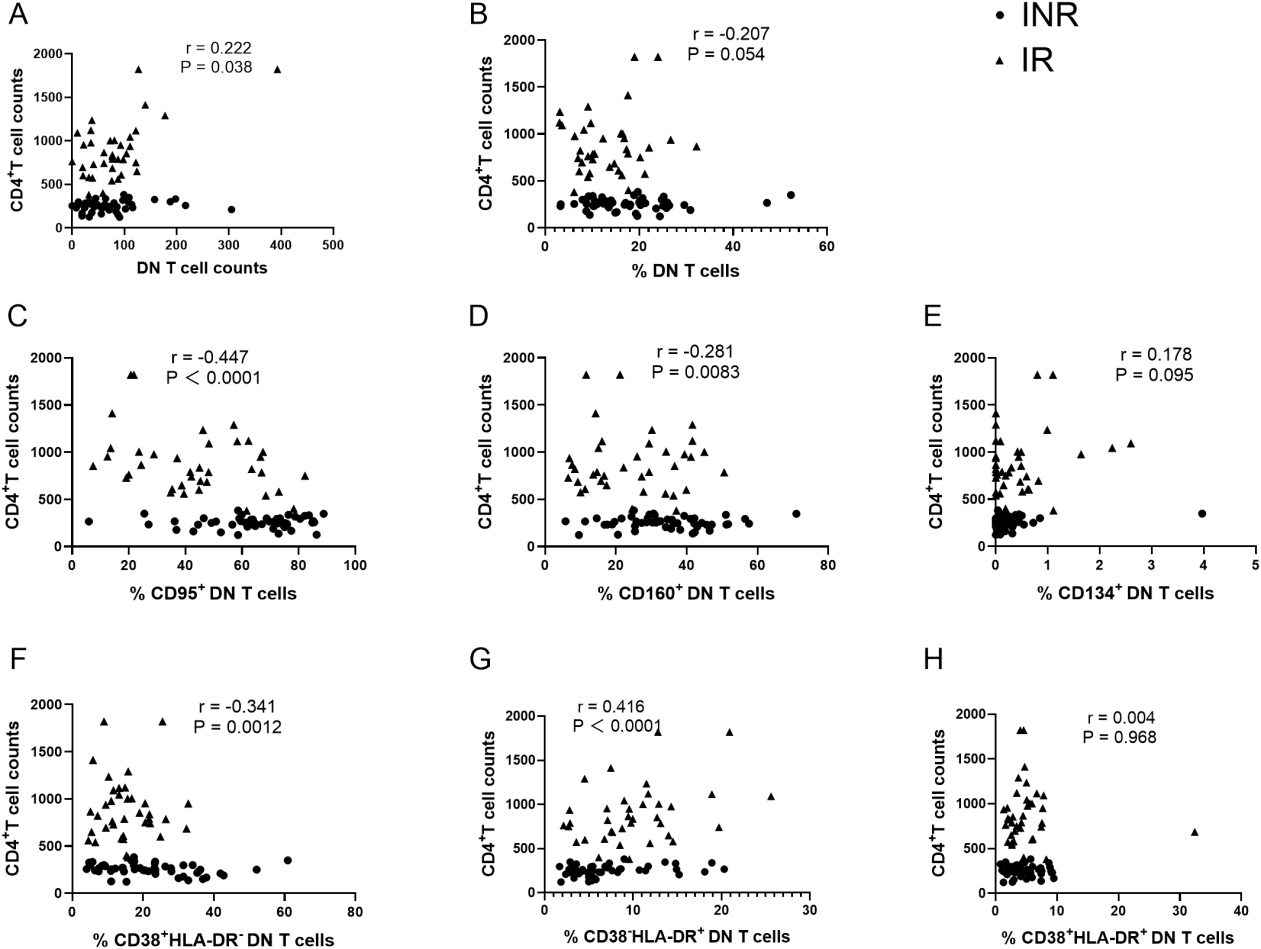
Associations between CD4^+^ T-cell counts and DN T cells and their phenotypic subsets. **(A, B)** Correlation of CD4^+^ T-cell counts with DN T-cell counts **(A)** and DN T-cell frequency **(B–E)** Correlation of CD4^+^ T-cell counts with the frequencies of CD95^+^, CD160^+^, and CD134^+^ DN T cells. **(F–H)** Correlation of CD4^+^ T-cell counts with CD38^+^HLA-DR^-^, CD38^-^HLA-DR^+^, and CD38^+^HLA-DR^+^ DN T cells. Each dot represents an individual participant (• INR; ▲ IR). Spearman correlation coefficients (r) and corresponding P values are shown. INR, immunological non-responders; IR, immunological responders; DN T cells, double-negative T cells.

To further evaluate the relationships between DN T-cell subsets and the key indicator of immune reconstitution (CD4^+^ T-cell counts), correlation analyses were performed using flow cytometric and clinical data from both groups. The absolute counts of DN T cells were positively correlated with CD4^+^ T-cell counts (r = 0.222, P = 0.038; **Figure 4A**), whereas the frequency of DN T cells showed a negative trend with CD4^+^ T-cell counts (r = −0.207, P = 0.054; **Figure 4B**). Further analyses demonstrated that the frequencies of CD95^+^ DN T cells and CD160^+^ DN T cells were both significantly negatively correlated with CD4^+^ T-cell counts (r = −0.447, P < 0.001 and r = −0.281, P = 0.008, respectively; **Figures 4C, D**). In contrast, no significant correlation was observed between the frequency of CD134^+^ DN T cells and CD4^+^ T-cell counts (**Figure 4E**). In addition, the frequency of CD38^+^HLA-DR^-^ DN T cells was negatively correlated with CD4^+^ T-cell counts (r = −0.341, P = 0.001; **Figure 4F**). Conversely, the frequency of CD38^-^HLA-DR^+^ DN T cells was positively correlated with CD4^+^ T-cell counts (r = 0.416, P < 0.001; **Figure 4G**). No significant correlation was observed between CD38^+^HLA-DR^+^ DN T cells and CD4^+^ T-cell counts (**Figure 4H**). To further examine the relationship between DN T-cell activation subsets and systemic immune activation, correlations between DN T-cell activation subsets and CD38^+^HLA-DR^+^CD8^+^ T cells were analyzed (**Supplementary Figure 1**). The frequency of CD38^+^HLA-DR^+^CD8^+^ T cells was positively correlated with CD38^+^HLA-DR^-^ DN T cells (r = 0.236, P = 0.028; **Supplementary Figure 1A**) and negatively correlated with CD38^-^HLA-DR^+^ DN T cells (r = −0.219, P = 0.041; **Supplementary Figure 1B**).

### 3.5 Altered phenotypic characteristics of the TCRαβ^+^ DN T-cell subset in PLWH with INR

**Figure 5:**
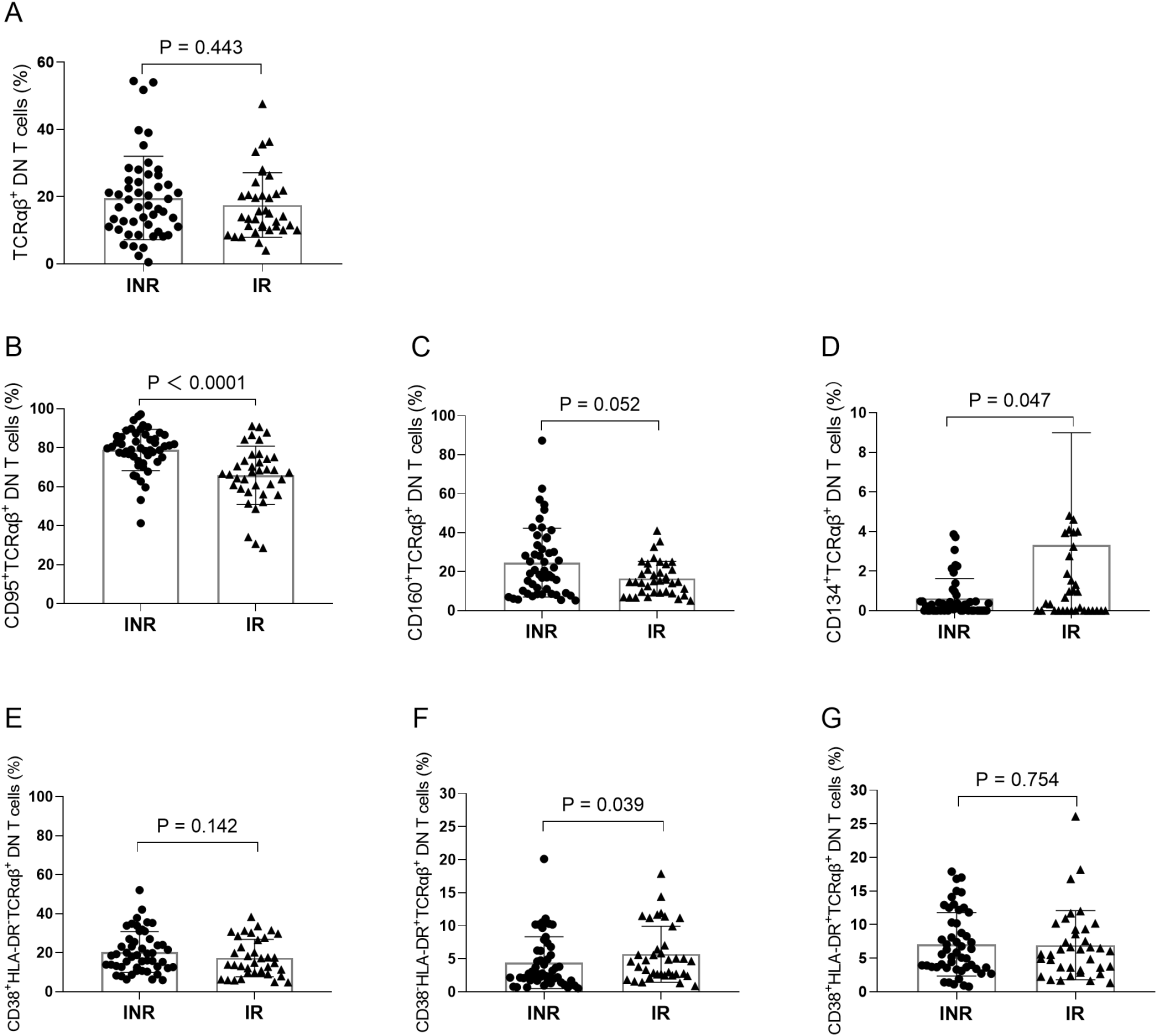
Phenotypic and activation characteristics of TCRαβ^+^ DN T cells in INR and IR individuals. **(A)** Frequency of TCRαβ^+^ DN T cells. **(B–D)** Frequencies of CD95^+^, CD160^+^, and CD134^+^ subsets within TCRαβ^+^ DN T cells. **(E–G)** Frequencies of CD38^+^HLA-DR^-^, CD38^-^HLA-DR^+^, and CD38^+^HLA-DR^+^ subsets within TCRαβ^+^ DN T cells. Each dot represents an individual participant (• INR; ▲ IR). Horizontal bars indicate the median with interquartile range (IQR). P values were calculated using the Mann–Whitney U test. INR, immunological non-responders; IR, immunological responders; DN T cells, double-negative T cells.

DN T cells represent a heterogeneous population, within which the TCRαβ^+^ subset has been reported to account for a substantial proportion. Therefore, subsequent analyses focused specifically on TCRαβ^+^ DN T cells. No significant difference was observed in the overall frequency of TCRαβ^+^ DN T cells between the INR and IR groups (P = 0.443; **Figure 5A**). However, the frequency of CD95^+^ TCRαβ^+^ DN T cells was significantly higher in the INR group than in the IR group (P < 0.001; **Figure 5B**). In contrast, the frequencies of CD134^+^ and CD38^-^HLA-DR^+^ TCRαβ^+^ DN T cells were significantly lower in the INR group than in the IR group (P = 0.047 and P = 0.039, respectively; **Figures 5D, F**). No significant differences were observed between the two groups in the frequencies of CD160^+^, CD38^+^HLA-DR^-^, or CD38^+^HLA-DR^+^ TCRαβ^+^ DN T cells (**Figures 5C, E, G**).

**Figure 6:**
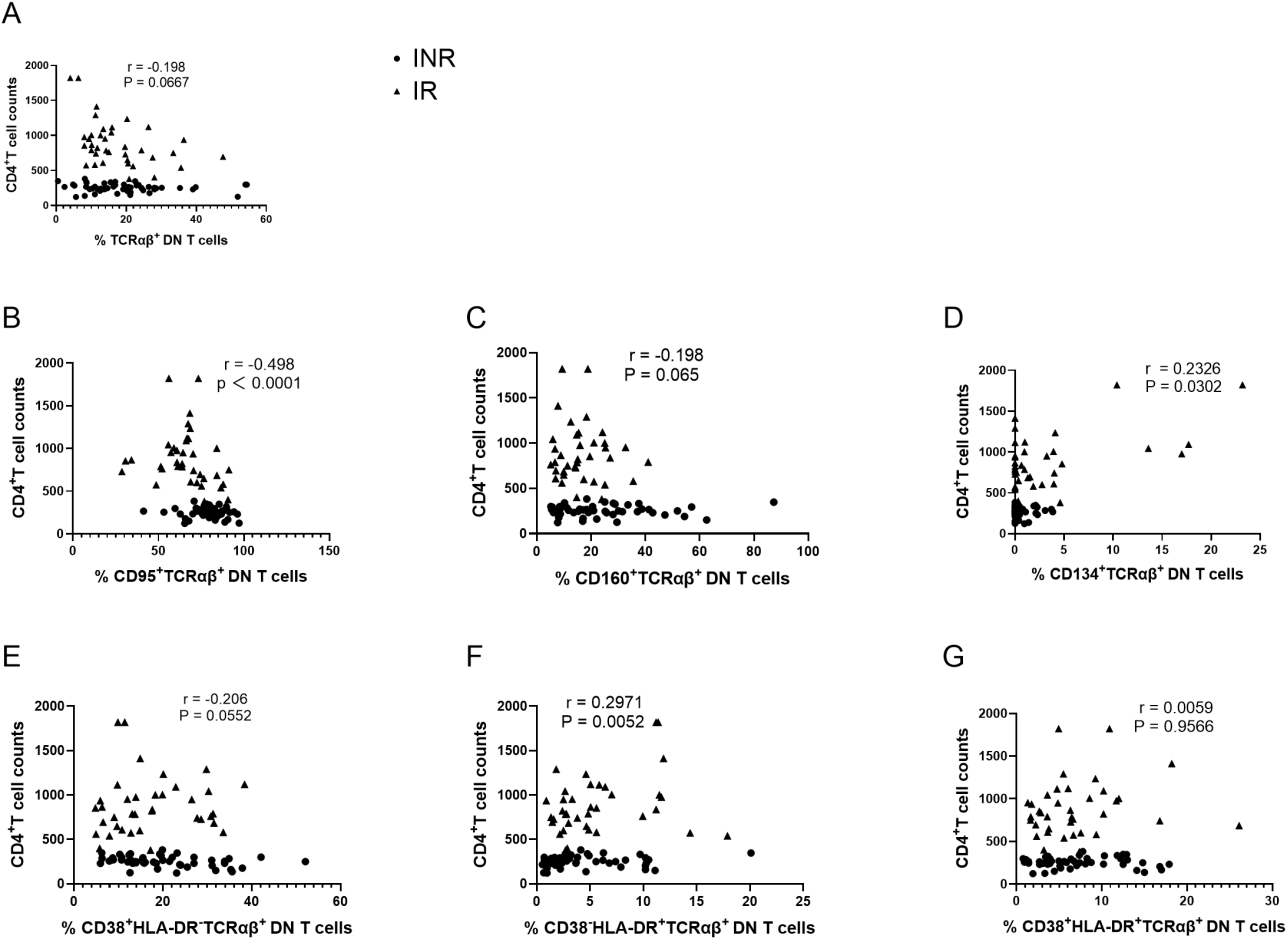
Associations between CD4^+^ T-cell counts and TCRαβ^+^ DN T cells and their phenotypic subsets. **(A)** Correlation of CD4^+^ T-cell counts with the frequency of TCRαβ^+^ DN T cells. **(B–D)** Correlation of CD4^+^ T-cell counts with the frequencies of CD95^+^, CD160^+^, and CD134^+^ subsets within TCRαβ^+^ DN T cells. **(E–G)** Correlation of CD4^+^ T-cell counts with CD38^+^HLA-DR^-^, CD38^-^HLA-DR^+^, and CD38^+^HLA-DR^+^ subsets within TCRαβ^+^ DN T cells. Each dot represents an individual participant (• INR; ▲ IR). Spearman correlation coefficients (r) and corresponding P values are shown. INR, immunological non-responders; IR, immunological responders; DN T cells, double-negative T cells.

To further examine the clinical relevance of these findings, correlation analyses were performed. The frequency of CD95^+^ TCRαβ^+^ DN T cells showed a negative correlation with CD4^+^ T-cell counts (r = −0.498, P < 0.001; **Figure 6B**). In addition, the frequencies of CD134^+^ and CD38^-^HLA-DR^+^ TCRαβ^+^ DN T cells were positively correlated with CD4^+^ T-cell counts (r = 0.233, P = 0.0302; **Figure 6D**; r = 0.297, P = 0.0052; **Figure 6F**).

### 3.6 CD95^+^ and CD160^+^ DN T cells are independently associated with INR

To further evaluate the associations between DN T-cell phenotypic parameters and INR, univariable and multivariate logistic regression analyses were performed. In univariable analyses, shorter treatment duration, older age, lower baseline CD4^+^ T-cell count, and higher frequencies of total DN T cells, CD95^+^ DN T cells, and CD160^+^ DN T cells were associated with INR. In multivariate analyses, treatment duration, age, and baseline CD4^+^ T-cell count were included as covariates to account for their potential confounding effects on immune reconstitution. A stepwise modeling strategy was applied, with each model including one DN T-cell phenotypic variable together with the predefined covariates. After adjustment, each 10% increase in the frequency of CD95^+^ DN T cells was associated with higher odds of INR (OR = 2.373, 95% CI: 1.227-4.588), whereas each 10% increase in the frequency of CD160^+^ DN T cells was associated with increased odds of INR (OR = 7.773, 95% CI: 1.520-39.759). Both CD95^+^ DN T cells and CD160^+^ DN T cells remained independently associated with INR, whereas total DN T cells and TCRαβ^+^ DN T-cell-related subsets were not statistically significant after adjustment (**Table 2**).

**Table 2:** Logistic regression analysis of factors associated with INR.

Variables	INR (n = 50)	IR (n = 37)	Univariable			Multivariable	
			OR (95% CI)		P values	OR (95% CI)	P values
ART duration (days), n (%)							
< 2124	35 (56.0%)	8(21.6%)					
≥ 2124	15 (30.0%)	29(70.0%)	0.118 (0.044-0.318)		< 0.001		
Age, years, n (%)							
< 50	18 (36.0%)	25 (67.6%)					
≥ 50	32 (64.0%)	12(32.4%)	3.704 (1.508-9.096)		0.004		
Sex, n (%)							
Female	2 (4.0%)	4 (10.8%)					
Male	48 (96.0%)	33 (89.2%)	2.909 (0.503-16.813)		0.233		
Route of HIV acquisition, n (%)							
Homosexual	32 (64.0%)	26 (70.3%)					
Heterosexual	13 (26.0%)	8 (21.6%)	0.738 (0.161-3.383)		0.152		
Other	5 (10.0%)	3 (8.1%)	0.975 (0.182-5.235)		0.976		
Baseline CD4^+^ T-cell count (cells/µL), n (%)							
< 200	46 (92.0%)	2 (5.4%)					
≥ 200	4 (8.0%)	35 (94.6%)	0.005 (0.001-0.029)	< 0.001			
Baseline HIV viral load (copies/mL), n (%)							
VL < 105	27 (54.0%)	25 (67.6%)					
VL ≥ 105	18 (36.0%)	8 (21.6%)	2.083 (0.770-5.633)	0.148			
DN T/CD3^+^ T, median (IQR)	0.679 (0.581-0.847)	0.448 (0.240-0.620)	1.947 (1.055-3.593)	0.033		2.029 (0.499-8.254)	0.323
% CD95^+^ DN T, median (IQR)	0.324 (0.253-0.422)	0.246 (0.140-0.364)	1.749 (1.337-2.288)	< 0.001		2.373 (1.227-4.588)	0.010
% CD160^+^ DN T, median (IQR)	0.001 (0.001-0.005)	0.003 (0.000-0.006)	1.730 (1.200-2.493)	0.003		7.773 (1.520-39.759)	0.014
% CD95^+^ TCRαβ^+^ DN T, median (IQR)	0.198 (0.090-0.345)	0.148 (0.093-0.225)	2.344 (1.502-3.658)	< 0.001		1.855 (0.867-3.971)	0.111
% CD160^+^ TCRαβ^+^ DN T, median (IQR)	0.002 (0.000-0.006)	0.010 (0.000-0.040)	1.574 (1.081-2.291)	0.018		1.748 (0.939-3.255)	0.078

Data are presented as n (%) or median (IQR). INR, immunological non-responders; IR, immunological responders; ART, antiretroviral therapy; VL, viral load; DN T cells (double-negative T cells). Variables with P < 0.05 in univariable analysis were considered for multivariable logistic regression. Age, ART duration, and baseline CD4^+^ T-cell count were treated as confounders and included in all multivariable models. A separate-model approach was applied in which each DN T-cell-related parameter was entered individually together with these covariates to evaluate its independent association with INR. Therefore, only DN T-cell-related variables are presented in the multivariable column.

**Figure 7:**
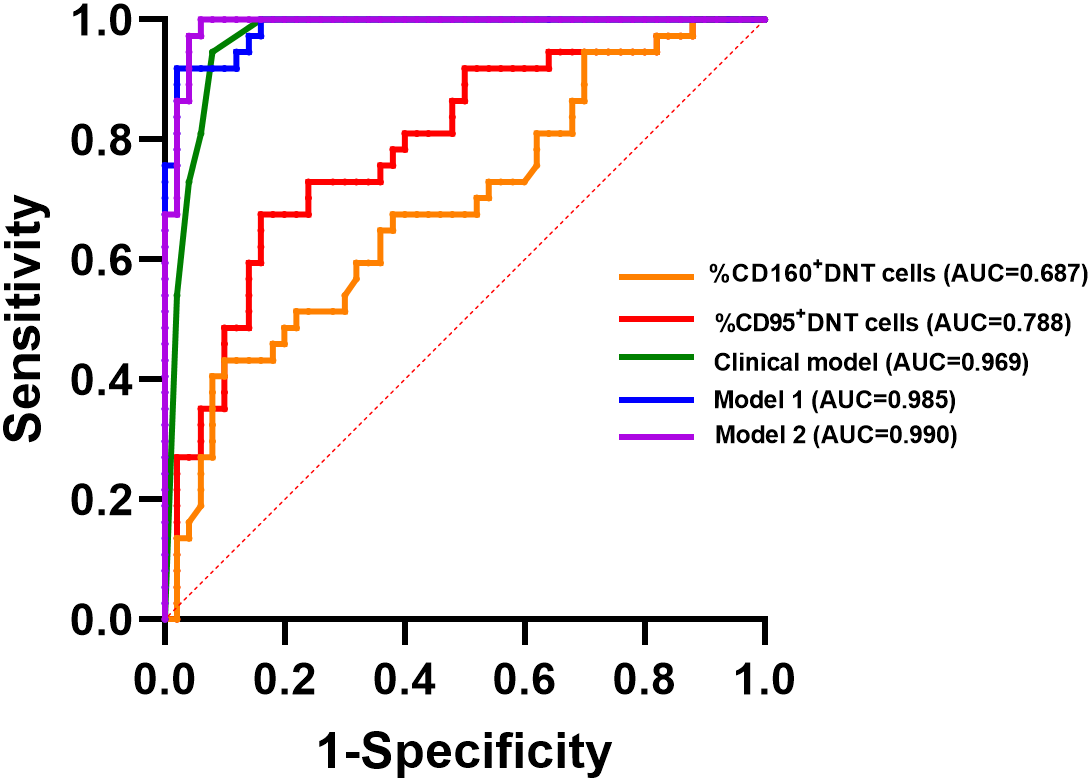
Receiver operating characteristic (ROC) curve analysis evaluating the discriminatory ability of DN T-cell phenotypes for distinguishing INR and IR individuals. ROC curves were generated for %CD160^+^ DN T cells, %CD95^+^ DN T cells, the clinical model (including ART duration, baseline CD4^+^ T-cell count, and age), Model 1 (clinical model plus %CD95^+^ DN T cells), and Model 2 (clinical model plus %CD160^+^ DN T cells). INR, immunological non-responders; IR, immunological responders; DN T cells, double-negative T cells.

ROC curve analyses were performed to further assess the ability of these parameters to discriminate INR (**Figure 7**). Among individual markers, CD95^+^ DN T cells showed AUC of 0.788 (95% CI: 0.691-0.885), with an optimal cutoff value of 3.5, yielding a sensitivity of 94.0% and a specificity of 32.4%. CD160^+^ DN T cells showed a lower AUC of 0.687 (95% CI: 0.574-0.800), with an optimal cutoff value of 2.0 (sensitivity 84.0%, specificity 43.2%). The baseline model including age, treatment duration, and baseline CD4^+^ T-cell count showed an AUC of 0.969 (95% CI: 0.934-1.000). After the addition of CD95^+^ DN T cells or CD160^+^ DN T cells, the AUC increased to 0.985 (95% CI: 0.968-1.000) and 0.990 (95% CI: 0.975-1.000), respectively.

## 4 Discussion

Through an integrated analysis of ART-treated PLWH, our findings reveal that individuals with INR exhibit increased systemic immune activation and a higher proportion of circulating DN T cells. Elevated expression of the apoptosis-related receptor CD95 and the co-inhibitory molecule CD160 on DN T cells was negatively correlated with CD4^+^ T-cell counts and independently associated with INR in multivariate analyses. These findings extend previous studies that primarily focused on quantitative changes by providing a phenotypic characterization of DN T cells in INR. Notably, we identified an independent association between CD95^+^ and CD160^+^ DN T-cell subsets and INR, highlighting their potential as immunological indicators of immune dysfunction. Consistent with these findings, ROC analyses demonstrated the ability of these phenotypes to discriminate between INR and IR, suggesting that CD95^+^ and CD160^+^ DN T cells could serve as cellular markers reflecting immune dysregulation in ART-treated PLWH.

We observed that the frequency of peripheral DN T cells was significantly higher in individuals with INR than in those with IR. This finding differs from previous findings by Lu et al., who described reductions in both the frequency and absolute number of DN T cells in INR (19). Such discrepancies may reflect differences in baseline immune status, timing or regimens of ART initiation, or gating strategies. Previous studies have also indicated that DN T-cell dynamics during chronic viral infection and persistent immune activation can be highly heterogeneous and influenced by phenotypic definitions and analytical approaches (20). Notably, in our cohort, absolute DN T-cell counts were positively correlated with CD4^+^ T-cell counts, whereas DN T-cell frequency showed an inverse trend. Because DN T-cell frequency is calculated relative to the total T-cell population, substantial CD4^+^ T-cell depletion in INR may lead to a relative increase in the proportional representation of DN T cells even when their absolute numbers do not increase. This”count-frequency dissociation”therefore likely reflects a compositional shift within the T-cell compartment rather than a true expansion of the DN T-cell population. In addition, chronic antigenic stimulation and sustained immune activation may promote the conversion of CD4^+^ or CD8^+^ T cells into DN T cells, potentially contributing to the increased DN T-cell frequency observed in peripheral blood (21–23). Overall, the elevated DN T-cell frequency in INR likely reflects altered T-cell subset composition associated with CD4^+^ T-cell depletion and persistent immune activation rather than true expansion. These findings suggest that DN T-cell frequency or absolute counts alone may not fully reflect their immunological relevance, highlighting the importance of phenotypic characterization.

During phenotypic characterization of DN T cells, we identified coordinated increases in CD95 and CD160 expression on DN T cells in INR. CD95 (Fas), a key receptor involved in extrinsic apoptotic signaling, was increased on DN T cells in the INR group. This finding is consistent with previous reports of persistent activation of apoptosis-related pathways during chronic HIV infection (24, 25) and may reflect apoptosis-associated phenotypic alterations in DN T cells. However, because FasL expression and functional assays were not assessed in the present study, it remains unclear whether elevated CD95 expression on DN T cells directly contributes to CD4^+^ T-cell apoptosis or instead reflects a broader state of immune dysregulation. Similarly, CD160 has been associated in previous studies with altered functional states of CD8^+^ T cells during chronic HIV infection (10, 12), but its expression on DN T cells, particularly in the context of INR, has not been well characterized. In our study, increased CD160 expression on DN T cells was observed in INR and was negatively correlated with CD4^+^ T-cell counts. However, functional assays were not performed, and therefore the biological significance of CD160^+^ DN T cells cannot be determined. The concurrent increase in CD95 and CD160 expression on DN T cells, together with elevated CD38^+^HLA-DR^+^CD8^+^ T-cell frequency, suggests an association between systemic immune activation and phenotypic remodeling of DN T cells in INR. In addition, no significant difference in CD134 expression on DN T cells was observed between the INR and IR groups. CD134 (OX40) is a costimulatory receptor that is typically upregulated following antigen stimulation and T-cell activation, whereas its expression on resting peripheral T cells is generally low. As PBMCs were analyzed *ex vivo* without *in vitro* stimulation, the lack of differential CD134 expression may reflect limited activation-induced upregulation under resting conditions rather than absence of biological relevance of this pathway (26, 27).

We further evaluated the activation status of DN T cells. We observed that the frequency of CD38^-^HLA-DR^+^ DN T cells was positively correlated with CD4^+^ T-cell counts and was relatively reduced in INR. This subset may represent a relatively mature activation phenotype (28, 29). In contrast, CD38-expressing DN T-cell subsets, including CD38^+^HLA-DR^-^ DN T cells (activation-associated phenotype) and CD38^+^HLA-DR^+^ DN T cells (highly or persistently activated phenotype) (30, 31), were relatively expanded in INR and both showed negative correlations with CD4^+^ T-cell counts. These findings indicate a shift toward more activated DN T-cell phenotypes in INR. Persistent immune activation has been widely reported in ART-treated PLWH despite viral suppression (32, 33). Such persistent activation may contribute to the altered activation profiles of DN T cells observed in this study. Moreover, the observed associations between CD38^+^HLA-DR^+^CD8^+^ T cells and activated DN T-cell subsets further support a link between systemic immune activation and DN T-cell phenotypic remodeling. Together, these findings suggest that DN T cells in INR exhibit coordinated activation-associated phenotypic changes, which may reflect immune dysregulation. However, the functional implications of these alterations require further investigation.

In addition, we performed a focused analysis of the TCRαβ^+^ DN T-cell subset, which represents a major component of peripheral DN T cells and has been implicated in immune regulation. Previous studies have reported that increased frequencies of TCRαβ^+^ DN T cells in PLWH are associated with the production of anti-inflammatory cytokines such as IL-10 and TGF-β, or with Fas/FasL-mediated induction of effector T-cell apoptosis, processes that may contribute to limiting excessive immune activation (34). In our cohort, CD95 expression on TCRαβ^+^ DN T cells was significantly elevated in INR and showed a negative correlation with CD4^+^ T-cell counts. This finding is consistent with the apoptosis-associated phenotype observed in total DN T cells. Elevated CD95 expression may indicate increased susceptibility to apoptosis in this subset. However, because functional assays were not performed, the precise functional consequences of this phenotype remain to be determined. By contrast, higher frequencies of CD134^+^ and CD38^-^HLA-DR^+^ TCRαβ^+^ DN T cells were observed in the IR group, suggesting a more regulated activation phenotype in individuals with immune reconstitution. Overall, these findings suggest that phenotypic remodeling of the TCRαβ^+^ DN T-cell subset may be associated with immune dysregulation in INR.

Finally, we evaluated the associations between DN T-cell phenotypes and INR using multivariable logistic regression analysis. Increased frequencies of CD95^+^ DN T cells and CD160^+^ DN T cells were independently associated with INR after adjustment for age, ART duration, and baseline CD4^+^ T-cell counts. These findings suggest that DN T-cell phenotypic alterations are associated with the immunological features of INR beyond baseline immunosuppression. Receiver operating characteristic analyses showed that CD95^+^ DN T cells exhibited moderate discriminatory ability for distinguishing INR from IR, with relatively high sensitivity. In contrast, CD160^+^ DN T cells demonstrated weaker discriminatory performance. Although the incremental improvement beyond conventional clinical variables was modest, DN T-cell phenotypes may provide complementary information on immune functional status. Compared with conventional indicators based on CD4^+^ T-cell counts (35), DN T-cell phenotyping may provide additional insight into immune alterations. In particular, CD95 and CD160 expression may reflect phenotypic features related to apoptosis-associated and inhibitory signaling. However, because the present study is cross-sectional and functional assays were not performed, the biological implications of these phenotypes remain to be clarified. Therefore, these findings should be interpreted cautiously given the cross-sectional design and limited sample size. Future longitudinal and functional studies will be required to determine the temporal dynamics and biological significance of these DN T-cell phenotypes.

In summary, this study demonstrates that DN T-cell phenotypic alterations, particularly increased CD95 and CD160 expression, are associated with immune activation and incomplete immune reconstitution in ART-treated PLWH.However, these findings should be interpreted cautiously due to the cross-sectional design, lack of functional assays, and relatively small sample size.Future longitudinal and functional studies are needed to further clarify the temporal dynamics and biological significance of DN T-cell phenotypes during immune reconstitution.

## 5 Conclusion

This study demonstrates that DN T cells in PLWH with incomplete immune reconstitution exhibit phenotypic alterations characterized by increased CD95 and CD160 expression. Increased frequencies of CD95^+^ and CD160^+^ DN T cells were independently associated with INR and showed moderate discriminatory ability. These findings suggest that DN T-cell phenotypes may serve as indicators of immune dysregulation in ART-treated PLWH.

## Data Availability

The raw data supporting the conclusions of this article will be made available by the authors, without undue reservation.
