# Supplementary figures and images for: Increased CD95+ or CD160+ double-negative T-cell subsets are associated with incomplete immune reconstitution in ART-treated people living with HIV
Source: Front Immunol. 2026 Apr 16;17:1806055. doi: 10.3389/fimmu.2026.1806055 (PMC13128653; doi:10.3389/fimmu.2026.1806055)

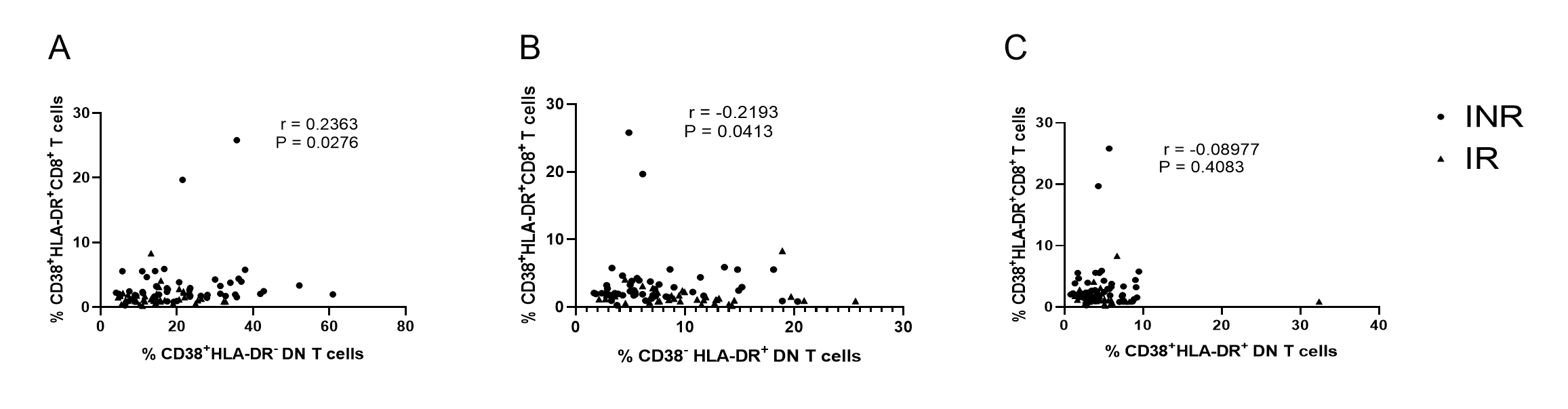

Supplement: Supplementary file 1 [file Image1.tif]
